# Arabic translation and validation of pediatric lower urinary tract symptom score (PLUTSS)

**DOI:** 10.1080/2090598X.2022.2108190

**Published:** 2022-08-11

**Authors:** Amr Al-Najar, Ibrahim Al-Nadhari, Sami Basabih, Fawaz Alobathani, Cem Akbal

**Affiliations:** a Faculty of Medical Sciences, Saba University, Sana’a, Yemen; b Department of Urology, Al-Wakra Hospital, HMC, Doha, Qatar; c Department of Urology, Al-Kuwait University Hospital, Sana’a, Yemen; d Department of Pediatrics, Al-Kuwait University Hospital, Sana’a, Yemen; e Department of Urology, Marmara University School of Medicine, Istanbul, Turkey

**Keywords:** Arabic validation, lower urinary tract symptoms, Pediatric Lower Urinary Tract Symptom Score

## Abstract

**Objectives:**

To develop and validate an Arabic version of the pediatric lower urinary tract symptom score (PLUTSS).

**Methods:**

The linguistic translation of the PLUTSS into Arabic was carried out by following the guidelines that have been set out for cross-cultural adaptation of health-related QoL measures (Translation, Reconciliation, Retranslation, Review of retranslation, Debriefing and final review). The questionnaires were applied to 80 patients, 40 patients seeking urology clinic for lower urinary tract symptoms (LUTS) and 40 patients visiting a pediatric clinic without urological compliant. The discrimination validity and strength of association were tested using Mann-Whitney and chi-square tests. Reliability of translation was tested for internal consistency using the Cronbach’s α and ROC Curve was used to evaluate the ability of the questionnaire to discriminate between cases and controls.

**Results:**

Patients with LUTS had a higher PLUTSS score and QoL score than controls (P < 0.001). The value of Cronbach’s alpha of the 13 items (excluding Qol) evaluated on the scale was 0.717 (95% CI: 0.616–0.800). The ROC curve determined the ability of the questionnaire to discriminate between cases and controls where the area under the curve was 0.901 (95% CI: 0.830–0.972).

**Conclusion:**

The Arabic translated version of the PLUTSS is an acceptable and reliable tool for assessing and evaluating pediatric patients with LUTS in Arabic-speaking countries.

## Introduction

Lower urinary tract symptoms (LUTS) in children are causing a huge burden on the health system to any society as its prevalence had been reported in values between 10% and 75% depending on the population studied [1–3]. The International Children’s Continence Society (ICCS), in an attempt to reach a global uniformity and clarity in characterizing lower urinary tract dysfunction, reported the standard terminology of lower urinary tract function in children and adolescents [4].

Simple diagnostic tools were required to evaluate and screen children for LUTS. Accordingly, a number of questionnaires to evaluate, screen and monitor the response to treatment in children with lower urinary tract symptoms were developed and validated [5–7]. These questionnaires are a highly effective method for evaluating patients, the impact of the dysfunction on the quality of life of patients and their parents, and they also aid in assessing the patient’s response to the treatment. Many of these questionnaires had been validated in its original language or linguistically translated and validated in many languages. Despite the fact that Arabic is being spoken by more than 420 million people in the Arab World and is recognized by the United Nations as one of the six official Languages, none of these questionnaires designed for pediatric lower urinary tract symptoms were translated to Arabic Language and validated. One of the developed questionnaires, the Pediatric Lower Urinary Tract Symptom Score (PLUTSS) had been proved to be effective in screening and monitoring the response of children to treatment of overactive bladder [6]. To date and to the best of our knowledge, there are currently no validated Arabic translations of PLUTSS questionnaire.

We aimed at translating the PLUTSS to Arabic and validating this Arabic version of the PLUTSS so that it can be utilized in Arabic-speaking countries for the assessment and monitoring of children with lower urinary tract symptoms.

## Materials and methods

### Steps of translation

The linguistic translation of the PLUTSS into Arabic was carried out by following the guidelines that have been set out for cross-cultural adaptation of health-related QoL measures. The following steps were carried out during the translation process:

a. Preparation and permission: Dr. Cem Akbal, author of the original work, was asked for his permission to carry out the cultural adaptation of the scale validated by them.

b. Translation: two translations of the original scale into Arabic were made by two bilingual translators to obtain a version by consensus. The translators are professors in the field of English translation studies, and were provided the appropriate instructions to carry out the translation.

c. Reconciliation: the two translators reviewed both translations and produced a third version due to reconciliation of the previous two.

d. Retranslation: the reconciled version was again retranslated into English.

e. Review of the retranslation: a comparison was made between the original version and the retranslation into English to assess inconsistencies or important discrepancies.

f. Debriefing or pre-test: the new scale was applied as a pilot test to 10 parents with the same population characteristics to determine the degree of understanding of the questions. They rated them as: understandable or not understandable; and the motive was determined. Subsequently, the relevant changes were made.

g. Final review: the entire process was reviewed by the authors before applying it to the study and the final version was made.

### Study design

The present study was cross-sectional in nature, which was conducted at Al-Kuwait University Hospital, Sana’a, Yemen. The study has been approved by Saba University research committee. In this study, group A comprised of 40 paediatric patients who were accompanied with their parents and were presented to the urology clinic with a history of lower urinary tract symptoms. They were interviewed and the translated Arabic PLUTSS questionnaire was to be filled by the parent on behalf of their children, after they consented and agreed in the participation of the study. On the other hand, Group B comprised 40 children who had no current or past history of lower urinary tract symptoms and attended the paediatric clinics for other complaints and were used as control group. Children with neurogenic bladder, spina bifida or lower urinary tract abnormalities were excluded from the study.

The translated Arabic PLUTSS has 13 questions addressing lower urinary tract symptoms and one question for quality of life (QoL). Each question has an assigned value with maximum score of 35 and a cut-off point of 8.5 to distinguish between healthy and sick child [6].

### Statistical analysis

The data were extracted from the questionnaires and the statistical analysis was done using the statistical packages SPSS 22.0 (SPSS Inc. Chicago, IL) and Epi-info (Centers for Disease Control and Prevention, Atlanta, GA) software. The discrimination validity was evaluated by comparing the scores of cases with those of controls using the Mann–Whitney test. The chi-square test was used to determine the strength of association by comparing the percentage of patients with a score >8.5 in cases and controls using the established cut-off point of 8.5 points. The reliability of translation was tested for internal consistency using Cronbach’s α for each item of the score with accepted value of >0.7. Receiver operating characteristic curve (ROC curve), the area under the curve and its 95% confidence interval were used to evaluate the ability of the questionnaire to discriminate between cases and controls. The sensitivity and specificity for different cut-off points of the total score of the questionnaire were calculated. Further validity using the Spearman rank correlation coefficient was assessed by determining the correlation between the PLUTSS scores and the QoL question. Logistic regression analysis was used to assess the impact of the different predictors on the total score value. All p values presented were two-tailed, and p values <0.05 were considered as statistically significant.

## Results

The mean age was 9.28 (SD = 3.040); for cases was 10.33 (SD = 3.277) and for control was 8.23 (SD = 2.391), (P = 0.002). Regarding gender, 46.2% were male and 53.8% were female; for cases 57.5% were males and 42.5% were female; for the control 50.0% were males and 50.0% were female (P = 0.501).

The case group with LUTS has a higher PLUTSS and QoL score than the control group (P < 0.001 in both). The mean of the total PLUTSS, excluding question 14-which pertains to the QoL, was 17.41 (SD = 13.50); Here, the mean of total PLUTSS of the cases group was 28.70 (SD = 14.25), and the mean of total PLUTSS for the control group was 6.13 (SD = 6.00). The percentage of positive responses for each question in patients and control subjects are shown in Table 1.

**Table 1.** Showed the percentage of positive responses for each question in patients and control subjects.

Table 1	Cases, n (%)	Controls, n (%)	*P*
	(n = 40)	(n = 40)	
1. Does your child have urinary incontinence (peeing while not on the toilet) during the day?			*P* < 0.001
No	17 (42.5%)	28 (70.0%)	
Sometimes	5 (12.5%)	12 (30.0%)	
1–2 times/day	5 (12.5%)	0 (0.0%)	
Always	13 (32.5%)	0 (0.0%)	
2. If Yes to Question 2			*P* = 0.051
Nothing	17 (42.5%)	28 (70.0%)	
Wet underwear	2 (5.0%)	3 (7.5%)	
Wet underwear and pants	8 (20.0%)	4 (10.0%)	
Soaked clothes	13 (32.5%)	5 (12.5%)	
3. Does your child have urinary incontinence (peeing while not on the toilet) during the night?			*P* < 0.001
No	8 (20.0%)	28 (70.0%)	
1–2 nights/week	3 (7.5%)	8 (20.0%)	
3–5 nights/week	4 (10.0%)	2 (5.0%)	
6–7 nights/week	25 (62.5%)	2 (5.0%)	
4. If Yes to Question 4 …			*P* < 0.001
Nothing	8 (20.0%)	28 (70.0%)	
Wet sheets	6 (15.0%)	5 (12.5%)	
Soaked sheets	26 (65.0%)	7 (17.5%)	
5. My child goes to the toilet to pee …			*P* = 0.633
<7 times/day	26 (65.0%)	28 (70.0%)	
≥7 times/day	14 (35.0%)	12 (30.0%)	
6. My child has to strain to pee.			*P* = 0.108
No	28 (70.0%)	34 (85.0%)	
Yes	12 (30.0%)	6 (15.0%)	
7. My child experiences pain when s/he pees.			*P* = 0.592
No	32 (80.0%)	30 (75.0%)	
Yes	8 (20.0%)	10 (25.0%)	
8. My child pees intermittently when on the toilet.			*P* = 0.051
No	24 (60.0%)	32 (80.0%)	
Yes	16 (40.0%)	8 (20.0%)	
9. My child has to go to revisit the toilet to pee soon after s/he pees.			*P* = 0.130
No	31 (77.5%)	36 (90.0%)	
Yes	9 (22.5%)	4 (10.0%)	
10. My child has to run to the toilet when s/he feels the need to pee.			*P* = 0.793
No	31 (77.5%)	30 (75.0%)	
Yes	9 (22.5%)	10 (25.0%)	
11. My child can hold his/her pee by crossing his/her legs, squatting, or doing the ‘pee dance.’			*P* = 0.390
No	31 (77.5%)	34 (85.0%)	
Yes	9 (22.5%)	6 (15.0%)	
12. My child wets his/her clothes before reaching the toilet.			*P* = 0.044
No	16 (40.0%)	25 (62.5%)	
Yes	24 (60.0%)	15 (37.5%)	
13. My child does not pass stool every day.			*P* = 0.617
No	28 (70.0%)	30 (75.0%)	
Yes	12 (30.0%)	10 (25.0%)	
If your child experiences the symptoms mentioned above, does it affect his/her family, social or school life?			*P* < 0.001
Not at all	3 (7.5%)	25(62.5%)	
Sometimes	10 (25%)	11(27.5%)	
Seriously affects	27 (67.5%)	4 (10%)	

In the evaluation of internal consistency, the value of Cronbach’s alpha of the 13 items evaluated on the scale was 0.717 (95% CI: 0.616–0.800).

**Figure 1.**
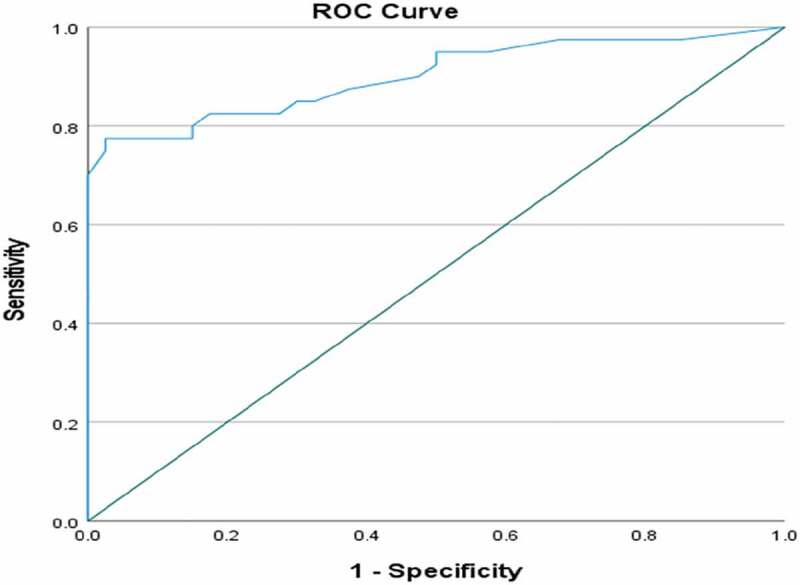
The ROC curve for the PLUSS score.

The ROC curve determined the ability of the questionnaire to discriminate between cases and controls (Figure 1) and the area under the curve was 0.901 (95% CI: 0.830–0.972). A sensitivity of 83% and a specificity of 75% were determined for a cut-off point in the total score of the questionnaire (without question 14) ≥10.5. These values were 85% and 70% for a cut-off point ≥8.5 points. At cut-off point of 12.5, the sensitivity and specificity were 82% and 80%, respectively.

**Figure 2.**
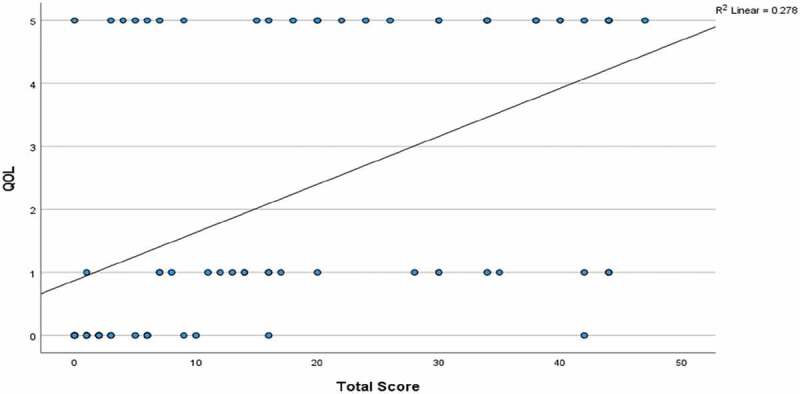
Scatter plot showing the correlation between the total score and QoL.

**Figure 3.**
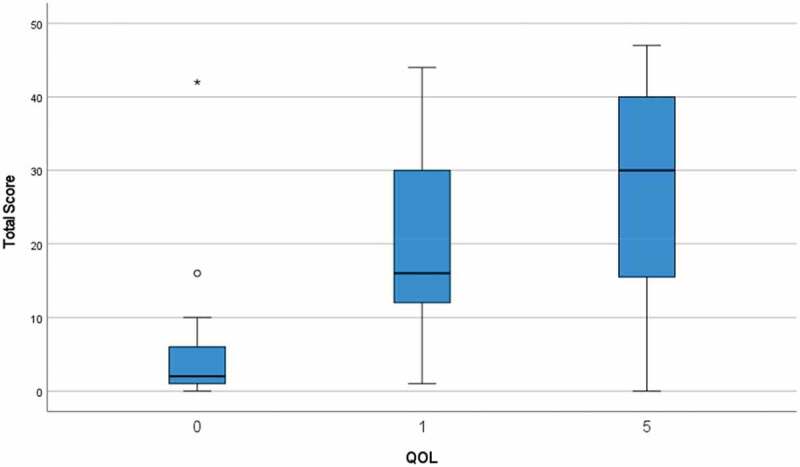
Box plot showing the total PLUSS score distribution with each scale of QoL.

The PLUTSS total score had a moderate and positive correlation with the QoL question (Spearman rank correlation coefficient, 0.53; P = 0.001). In addition, there was a low and positive correlation between age and QoL (Spearman rank correlation coefficient, 0.35; P = 0.002). The distribution of total PLUTSS with each scale of QoL is shown in Figures 2 and 3).

Logistic regression analysis was used to determine the independent variable (the cases and the control, gender) with total score >8.5 and the results are shown in Table 2.

**Table 2.** Showing the logistic regression analysis result for the independent variable (the cases and the control, gender) with the total score categories.

Independent variable		Total score (%)		Total (N)	Odd ratio	95% CI
		≤8.5	>8.5			
Gender	Female	37.8%	62.2%	37		
	Male	46.5%	53.5%	43	0.7	(0.3–1.7)
Status	Control	70%	30%	40		
	Cases	15%	85%	40	13.2	(4.4–39.7)

## Discussion

This study showed that the Arabic translated version PLUTSS questionnaire is a valid tool that allows the administrator to differentiate between children with LUTS and children without LUTS, with a sensitivity of ranging between 85% and 82% and specificity ranging between 83% and 70% depending on the cut-off point used. The values of the original questionnaire and its Spanish version were 90–100% and 90–95% at a cut-off point of 8.5, respectively (Sensitivity and Specificity) [6,8]. During the translation and validation stages, various steps were taken in order to ensure that the questionnaire is clear to the subjects and has strong internal consistency. In the translation stage, the steps that were carried out during the translation of the questionnaire were documented concisely, and the feedback that was provided from the groups involved in the study was also taken into consideration, and the recommended changes were made accordingly. This was done in order to ensure that a consensus was reached within the team before the questionnaire was distributed to the study subjects.

Our study confirmed that patient with lower urinary tract symptoms did have a high PLUTSS scores with a mean score of 28.70 (SD = 14.25) in the patients’ group and a mean of 6.13 (SD = 6.00) in the control group (p value <0.001). These results confirm the eligibility of the questionnaire which was previously reported to be a valid tool and its accuracy was superior to the ICIQ-CLUTS (International Consultation on Incontinence Questionnaire Pediatric Lower Urinary Tract Symptoms) when compared with the physician’s clinical impression [9].

During the process of validation, an internal consistency of 0.717 was calculated, which was 0.811 in the original version [6], 0.827 in the Spanish translated version [8] and 0.74 in the Persian translation [10].

The discriminant validity of the Arabic version revealed an area under the ROC curve of 0.901 (Figure 1), indicating that it correctly classifies a randomly selected child with lower urinary tract symptoms in 90% of the time. Similar values were recorded by the original version and Spanish translations with an area under the ROC curve of 0.962 and 0.998, respectively [6,8].

In the present study, all the measure variables were significant, both statistically and clinically, therefore indicating good internal consistency and high reliability of the Arabic version of PLUTSS. However, one of the main limitations of our present work is that standard Arabic language was used and tested in a single hospital (community). It will need to be tested and validated in different Arabic countries and communities as the Arabic dialect differ from one country to another.

## Conclusion

The Arabic version of the PLUTSS is a reliable and valid questionnaire which can be used in the evaluation and monitoring of pediatric patients with lower urinary tract symptoms in Arabic-speaking countries.

## Abbreviations

PLUTSS: Pediatric Lower Urinary Tract Symptom Score
